# Impact of CBCT frequency on target coverage and dose to the organs at risk in adjuvant breast cancer radiotherapy

**DOI:** 10.1038/s41598-021-96836-0

**Published:** 2021-08-30

**Authors:** Kai J. Borm, Yannis Junker, Mathias Düsberg, Michal Devečka, Stefan Münch, Hendrik Dapper, Markus Oechsner, Stephanie E. Combs

**Affiliations:** 1 Department of Radiation Oncology, Klinikum Rechts Der Isar, Medical School, Technical University Munich, Ismaningerstraße 22, 81675 Munich, Germany; 2 Deutsches Konsortium Für Translationale Krebsforschung (DKTK)-Partner Site Munich, Munich, Germany; 3 Institute of Radiation Medicine, Helmholtzzentrum München, Munich, Germany

**Keywords:** Breast cancer, Cancer imaging

## Abstract

The current study aims to assess the effect of cone beam computed tomography (CBCT) frequency during adjuvant breast cancer radiotherapy with simultaneous integrated boost (SIB) on target volume coverage and dose to the organs at risk (OAR). 50 breast cancer patients receiving either non-hypofractionated or hypofractionated radiotherapy after lumpectomy including a SIB to the tumor bed were selected for this study. All patients were treated in volumetric modulated arc therapy (VMAT) technique and underwent daily CBCT imaging. In order to estimate the delivered dose during the treatment, the applied fraction doses were recalculated on daily CBCT scans and accumulated using deformable image registration. Based on a total of 2440 dose recalculations, dose coverage in the clinical target volumes (CTV) and OAR was compared depending on the CBCT frequency. The estimated delivered dose (V95%) for breast-CTV and SIB-CTV was significantly lower than the planned dose distribution, irrespective of the CBCT-frequency. Between daily CBCT and CBCT on alternate days, no significant dose differences were found regarding V95% for both, breast-CTV and SIB-CTV. Dose distribution in the OAR was similar for both imaging protocols. Weekly CBCT though led to a significant decrease in dose coverage compared to daily CBCT and a small but significant dose increase in most OAR. Daily CBCT imaging might not be necessary to ensure adequate dose coverage in the target volumes while efficiently sparing the OAR during adjuvant breast cancer radiotherapy with SIB.

## Introduction

In recent years, significant progress has been made in adjuvant breast cancer radiotherapy, comprising the establishment of alternative fractionation schedules, more profound knowledge on partial breast irradiation and optimized target volume definition, particularly for the irradiation of the lymphatic drainage pathways^1–4^. In the meantime, the optimization of image guidance in modern breast cancer radiotherapy has been widely neglected. Increasingly complex target volumes and the use of modern irradiation techniques like VMAT and SIB demand sufficient position monitoring and correction more than ever. Despite its crucial impact on treatment accuracy and safety, image guided radiation therapy (IGRT) still lacks standardized guidelines and recommendations.

IGRT “employs imaging to maximize accuracy and precision throughout the entire process of treatment delivery”^5^, which is achieved by correcting the patient’s position prior to treatment delivery by aligning an online image to the planning CT. Online imaging can be implemented using either planar x-rays (2D), CBCT or surface scanners. Since the position of the breast is highly variable and the target volume is in close proximity to critical organs at risk, image guidance is widely used^6^. Previous studies on IGRT in breast cancer patients indicate that CBCT is superior or at least equivalent to 2D-based techniques regarding position correction^7–9^. In recent years, surface scanners are increasingly used as an alternative method of image guidance during breast cancer irradiation. However, according to recent analyses, CBCT remains the gold-standard as it leads to smaller deviations and enables a better spatial match with the planning CT^10,11^. These advantages of CBCT are particularly important in case of complex target volumes and treatment plans including simultaneous boost irradiation^12^.

A critical disadvantage of CBCT is the additional radiation exposure. According to a study by Quinn et al.^13^, daily CBCT imaging increases the dose to the contralateral breast, the contralateral lung and the heart by a relative 12%, 24% and 13%. As surface scanners are not yet available in most centers^6^, a reduction of CBCT frequency remains the most important course of action to reduce dose exposure. Furthermore, daily CBCT prolongs the treatment time and thereby reduces the daily treatment capacity of the facility, causing additional costs.

So far, the effect of CBCT frequency on target dose coverage and dose to the OAR in breast cancer patients remains mostly uncertain. Yet today, dose accumulation workflows based on non-rigid image registration enable precise estimations of the dose distribution depending on the CBCT frequency. This new approach bears the potential of solving the persisting issue of optimal CBCT frequency. The goal of the current study was to elaborate treatment recommendations regarding the use of CBCT in adjuvant breast cancer radiotherapy, aiming to keep the imaging dose as low as reasonably achievable.

## Methods

### Patients and radiotherapy

50 breast cancer patients receiving adjuvant radiotherapy with daily CBCT imaging (27–28 CBCT in 28 fractions (Fx) or 13–16 CBCT in 16 Fx) treated in our institute between 05/2016 and 09/2020 were included in the current analysis. Details regarding the CBCT protocol are summarized in Table 1. The same protocol was used for daily, weekly and EOD imaging.

**Table 1:** Details regarding the CBCT-protocol used for daily, EOD or weekly IGRT.

X-ray voltage (kVp)	110
X-ray current (mA)	20
X-ray millisecond (ms)	20
Gantry rotation (deg)	360
Number of projections	655
Exposure (mAs)	262
Fan type	Half fan
Bow-tie filter	Half
Default pixel matrix	384 × 384
Slice thickness (mm)	2.5
Scan time (min)	1.1

The patients gave informed consent for treatment and the study was approved by the local ethics committee (Technical University Munich, 103/21 S-EB). All patients received whole breast irradiation after lumpectomy, including a SIB to the tumor bed. 34 patients (69%) were additionally treated with regional lymph node irradiation (RNI) including the supra- and infraclavicular region ± the internal mammary region (n = 25, 50%). The prescribed dose to the whole breast was either 50.4 Gy in 28 Fx (n = 45) or 42.5 Gy in 16 Fx (n = 5). The prescribed dose to the tumor bed was either 58.8 Gy (n = 18) or 63 Gy (n = 27) in 28 Fx or 48 Gy in 16 Fx (n = 5). All treatment plans were created in Eclipse 15.6 (Varian Medical Systems, Palo Alto, CA, USA) treatment planning system (TPS) using VMAT technique. Contouring and treatment planning was performed according to current guidelines^3,14,15^ and all treatment plans were approved in house by a board of attending radiation oncologists prior to treatment delivery. The CTV to PTV margins were 10 mm with exclusion of lung tissue for the breast, 5 mm for the tumor bed and 5 mm for the lymph node areas. All patients received daily kV-CBCT imaging on a linear accelerator (Varian On Board Imager 1.6, Triology® or DHX®, Varian Medical Systems, Palo Alto, CA, USA) with online correction of the couch position along the x-, y- and z-axis (3D). Online registrations were verified by a radiation oncologist.

### Estimation of dose distribution based on CBCT images

**Figure 1:**
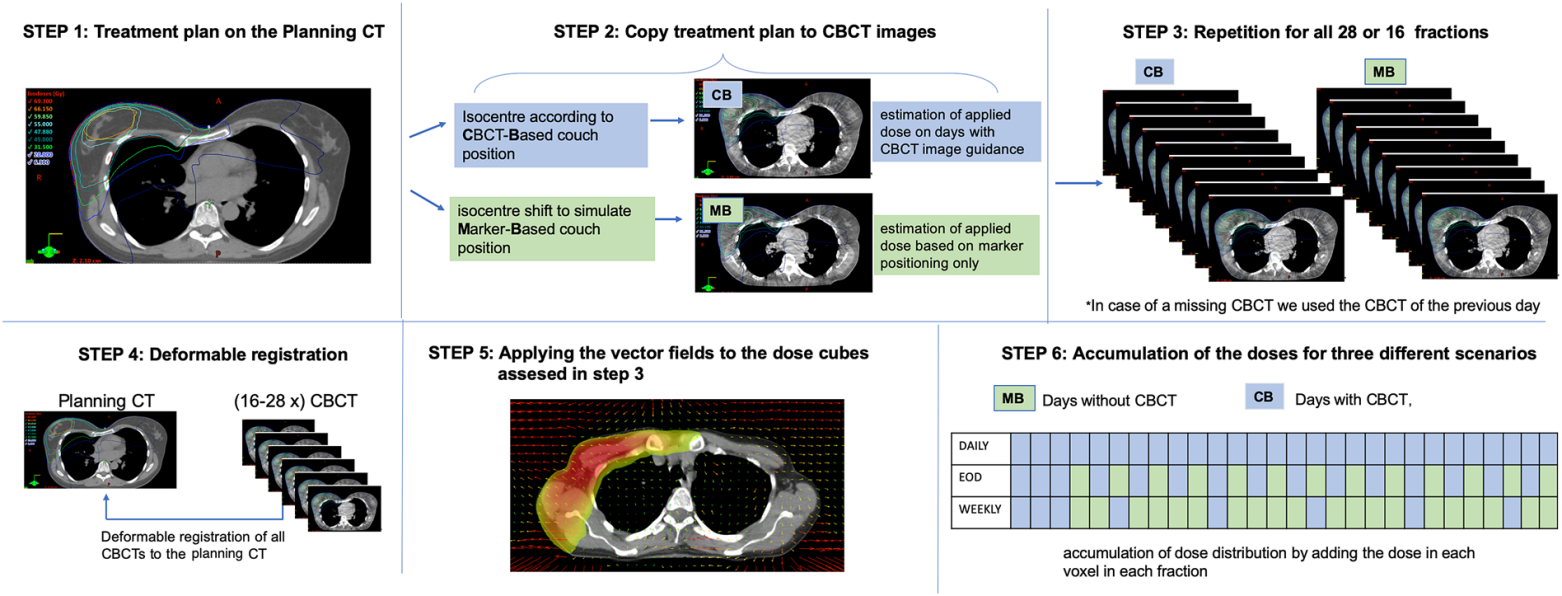
Workflow used to estimate the delivered dose in dependence of CBCT- frequency. *CB* conebeam-based positioning, *MB* marker-based positioning.

A dose accumulation workflow was implemented to estimate the dose distribution over all fractions (Fig. 1). For this, the applied plans were recalculated on each CBCT in Varian Eclipse 15.6, using a CBCT-site specific calibration curve. Deformable image registration (DIR) was utilized to calculate deformation vector fields (DVF), projecting each CBCT onto the corresponding planning CT. The acquired dose cubes and DVFs were then exported to an in-house written script, deforming and accumulating the doses for every fraction. The script was written in MATLAB2019b (The MathWorks Inc., Natick, MA, USA) and uses the image processing framework plastimatch 1.7.3 (The General Hospital Corporation Inc., Boston, MA, USA) for dose deformation and DICOM-RT import/export operations.

The Iso-Center configuration for the dose recalculations was set depending on the patient positioning method we intended to simulate. On days with CBCT-based positioning, we used the shifts from the online registrations (CB dose). In addition to the daily CBCT setup, we simulated two further setups, one comprising CBCT imaging every other day (EOD) and one comprising weekly CBCT imaging. On the remaining days, an optimized marker-based setup was simulated under the following 3 conditions:

1. CBCTs on days 1–3. The mean of those 3 CBCT-based shifts defines an optimized marker position.
2. On days without CBCT, this optimized marker position is used for patient positioning (MB dose).
3. The marker position is not adapted based on the acquired CBCT-scans throughout the treatment.

Based on this system, CB- and MB-doses were calculated and accumulated according to the imaging protocol in question, as illustrated under step 6 in Fig. 1. In total, 2440 CBCT dose recalculations were performed in order to evaluate the optimal IGRT-frequency.

### Dose evaluation

For dose evaluation, the accumulated dose cubes were reimported into the TPS and compared for dose coverage in the target volumes and dose to the OAR (heart, LAD, lung, contralateral breast). Whenever a structure was not completely captured by the field of view of the CBCT (e.g. lung in cranio-caudal direction) an *evaluation* structure was generated, comprising only the part of the structure that was fully delineated by the CBCT. Hereafter, the accumulated dose values were compared in dependence of the CBCT frequency (daily vs. EOD vs. weekly). Average dose-volume-histograms (DVH) were created for the target volumes and the OAR by calculating the mean volume of all patients receiving a dose from 0 to 70 Gy. To estimate statistical significance, we used a paired t test (normal distribution) and the Wilcoxon signed-rank (non-normal distribution). This pairwise test is sensitive to the existence of plan differences but independent of the magnitude of this difference. *P* values < 0.05 were considered statistically significant. Statistical analysis was not performed for the subgroup of patients treated with hypofractionated radiotherapy due to the small sample size (n = 5).

## Results

### Set-up errors

**Figure 2:**
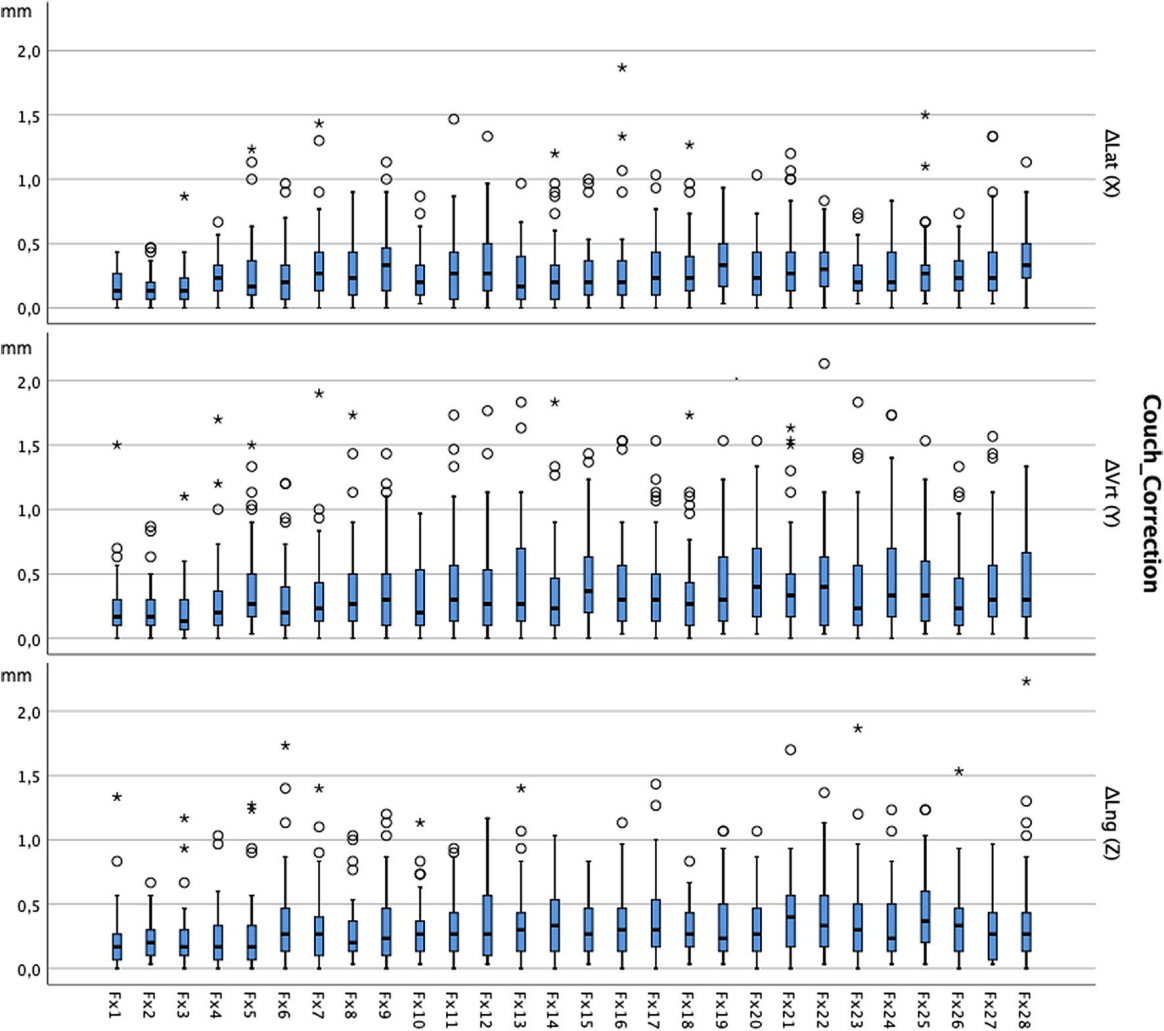
Deviation of marker-based position from CBCT-based position in the course of the treatment with 28 fractions. Median values (in mm) of all patients (n = 45) and 95% confidence interval (whiskers) treated with non-hypofractionated radiotherapy.

The median couch corrections (from the optimized marker-based position) after CBCT verification were 0.2 cm (0–1.9 cm) in lateral, 0.3 cm (0–2.1 cm) in cranio-caudal and 0.3 cm (0–3.2 cm) in anterior–posterior dimension. Figure 2 delineates the median couch-corrections of all patients undergoing non-hypofractionated radiotherapy. As can be seen in Fig. 2, the magnitude of couch-corrections after CBCT imaging increased during the treatment.

### Dose coverage in the CTV

The estimated delivered dose (V95%) for breast-CTV and SIB-CTV was significantly lower than the planned dose distribution, irrespective of the CBCT-frequency. Between daily CBCT and CBCT on EOD, no significant dose differences were found regarding V95% for both, breast-CTV and SIB-CTV. Weekly CBCT though led to a significant decrease in dose coverage in the CTV compared to daily CBCT. Nevertheless, the absolute difference regarding V95% was rather small (breast-CTV: Δ 0.6%; SIB-CTV: Δ 2.1%). A difference of mean dose of > 1 Gy between daily CBCT and CBCT on EOD (weekly CBCT) for the breast-CTV was observed in only 1 (2) patient(s). For the SIB-CTV, a dose difference > 1 Gy was observed in 2 (EOD) and 7 (weekly CBCT) patients, respectively.

**Figure 3:**
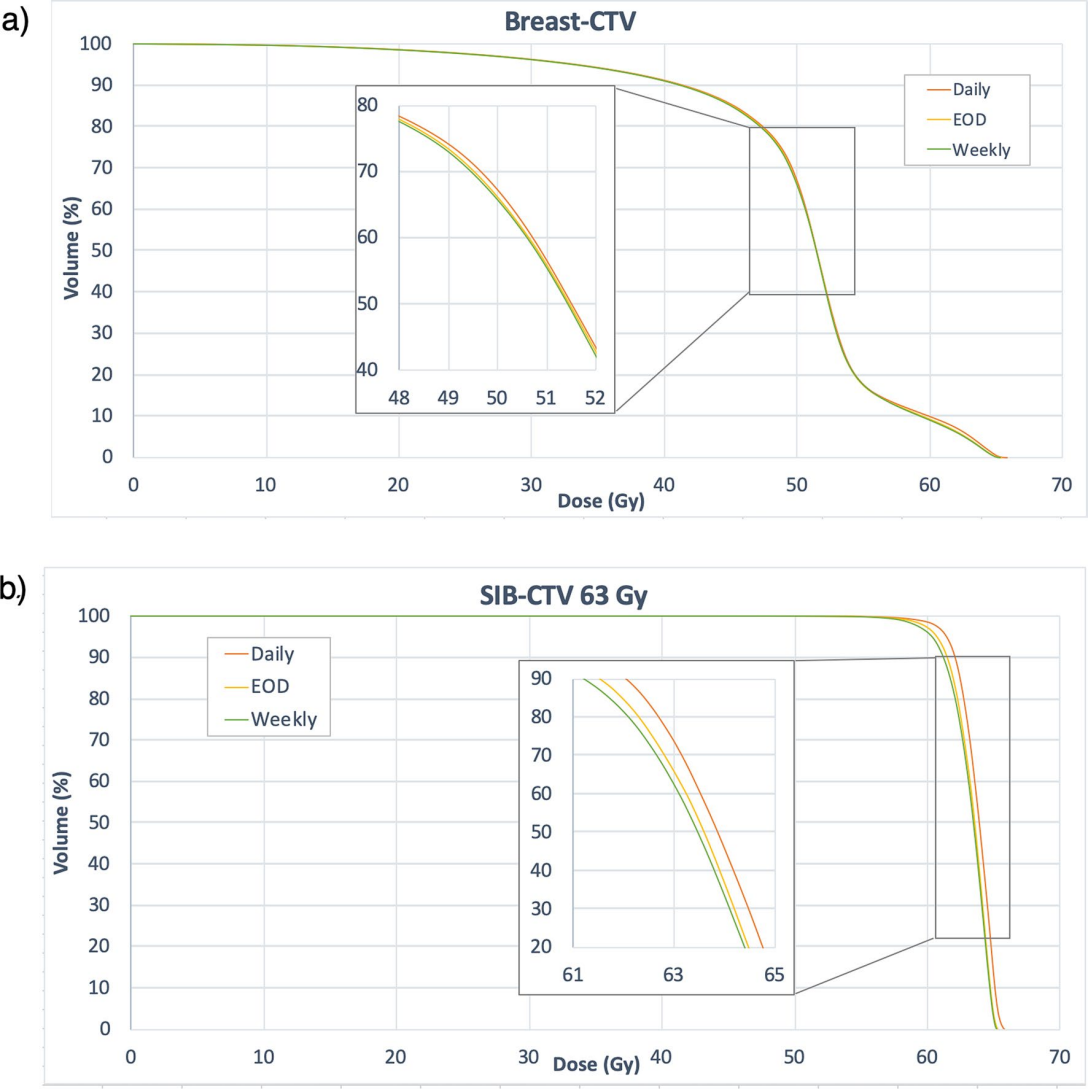
Dose volume histograms delineating the estimated delivered dose in the breast (**a**) and the SIB-CTV (**b**). Mean values of 28 patients with a prescribed dose to the Breast-PTV of 50.4 Gy and 63 Gy to the SIB-PTV.

In patients treated with hypofractionated radiotherapy (16 Fx), only very small dose differences were observed between the three different CBCT protocols (ΔDmean_max_: 0.2 Gy; ΔV95%_max_ 0.5%). The average values for Dmean and V95% in the breast-CTV and the SIB-CTV of all patients are summarized in Table 2. Figure 3 depicts the average DVHs of patients receiving non-hypofractionated radiotherapy and a SIB dose of 63 Gy (n = 27) in dependence of the CBCT frequency.

**Table 2:** Dose coverage of the breast-CTV and SIB-CTV and the in the planning CT compared and the estimated delivered dose distribution.

Non-hypofractionated radiotherapy (n = 45)	Planning-CT	Estimated delivered dose distribution		
		Daily CBCT	EOD	Weekly
**Breast-CTV**				
Dmean	51.4 ± 1.6 Gy	49.9 ± 2 Gy	49.8 ± 2.1 Gy	49.7 ± 2.1 Gy
			Daily vs. EOD Δ − 0.1 (− 2.1; 0.7) Gy *p* = 0.06	Daily vs. Weekly Δ − 0.2 (− 2.3; 1.0) Gy *p* = 0.01
V95%	85 ± 7.3%	77.4 ± 8.9%	77.1 ± 9.1%	76.8 ± 9.2%
			Daily vs. EOD Δ − 0.3 (− 6.9; 1.6) % *p* = 0.13	Daily vs. Weekly Δ0.6 (− 7.3; 2.2) % *p* = 0.02
**SIB-CTV**				
Dmean	61.8 ± 2.3 Gy	61.8 ± 2.4 Gy	61.5 ± 2.4 Gy	61.4 ± 2.4 Gy
			Daily vs. EOD Δ − 0.3 (− 1.9; 0.5) Gy *p* < *0.01*	Daily vs. Weekly Δ − 0.4 (− 2.1; 0.9) Gy *p* < *0.01*
V95%	98.6 ± 2.8%	97.0 ± 4.7%	96.1 ± 6.2%	94.9 ± 7.6%
			Daily vs. EOD Δ0.9(10.9; 1.2)% *p* = 0.07	Daily vs. Weekly Δ2.1(− 20.1; 2.1)% *p* < *0.01*

Hypofractionated radiotherapy (n = 5)	Planning-CT	Estimated delivered dose distribution		
**Breast-CTV**				
Dmean	40 ± 1.3 Gy	39.4 ± 1.3 Gy	39.4 ± 1.4 Gy	39.4 ± 1.4 Gy
V95%	89.2 ± 4.9%	83.5 ± 3.6%	83.4 ± 3.8%	83.3 ± 3.8%
**SIB-CTV**				
Dmean	47.2 ± 1.7 Gy	47.5 ± 2.4 Gy	47.5 ± 2.3 Gy	47.5 ± 2.3 Gy
V95%	99.8 ± 0.3%	97 ± 5.5%	96.9 ± 5.6%	96.5 ± 6.3%

Mean values and standard deviation. Mean absolute differences between CBCT-protocols (range).

### Dose in the OAR

For heart Dmean and V10Gy, no significant differences were found between daily CBCT and CBCT on EOD. Weekly CBCT, however, lead to a significantly higher heart Dmean compared to daily CBCT with an absolute difference of Δ0.15 Gy. V10Gy did not significantly differ between the three CBCT protocols. For the LAD, both CBCT on EOD and weekly CBCT resulted in significantly higher doses compared to daily CBCT, the absolute difference between mean values being Δ0.36 Gy. Interestingly, the maximum dose values for Dmean and V10Gy in the heart and LAD were lower in case of CBCT on EOD and weekly CBCT compared to daily CBCT.

**Figure 4:**
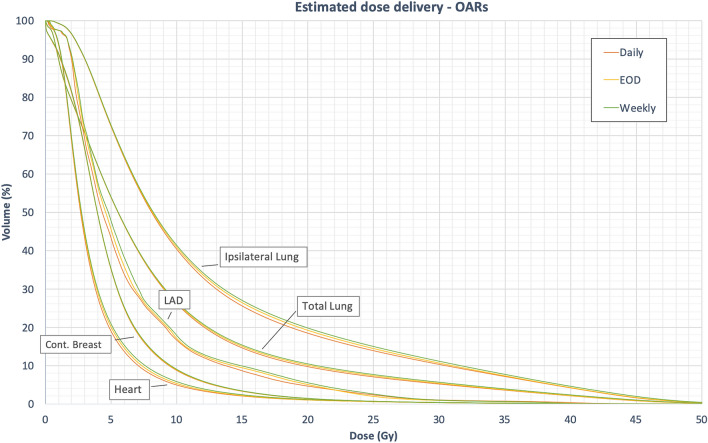
Dose volume histograms delineating the estimated delivered dose in the OARs. Mean values of 45 patients with a prescribed to the Breast-PTV of 50.4 Gy and 58.8 Gy or 63 Gy, respectively, to the SIB-PTV.

For the ipsilateral lung, there was no significant difference in Dmean between daily CBCT and CBCT on EOD, whereas weekly CBCT lead to significant difference (Δ0.4 Gy) compared to daily CBCT. For the total lung, Dmean did not significantly differ between the three CBCT protocols. V20Gy though was significantly higher using either CBCT on EOD or weekly CBCT. For the contralateral breast no significant differences were observed. The average dose values are summarized in Table 3. Figure 4 depicts the average DVHs of patients receiving non-hypofractionated radiotherapy, demonstrating the differences in dose distribution in dependence of the CBCT-frequency.

**Table 3:** Dose coverage in the OARs in the planning CT compared and the estimated delivered dose distribution in dependence of CBCT-frequency.

Non-hypofractionated radiotherapy (n = 45)	Planning-CT	Estimated delivered dose distribution		
		Daily CBCT	EOD	Weekly
**Heart**				
Dmean	4.2 ± 1.8 Gy	3.8 ± 1.9 Gy	3.8 ± 1.9 Gy	3.9 ± 1.9 Gy*
V10Gy	5.4 ± 7.8%	5 ± 8%	5.4 ± 8.2%	5.9 ± 8.5%
**LAD**				
Dmean	7 ± 4.1 Gy	6.5 ± 4.3 Gy	6.6 ± 4.2 Gy*	6.8 ± 4.3 Gy*
**Ipsi. lung**				
Dmean	13.2 ± 1.4 Gy	12.2 ± 1.5 Gy	12.4 ± 1.8 Gy	12.6 ± 2.1 Gy*
V20Gy	20.4 ± 2.1%	18.4 ± 3.1%	19.1 ± 4%*	19.7 ± 5%*
**Total lung**				
Dmean	9.1 ± 1.5 Gy	8.4 ± 1.4 Gy	8.5 ± 1.5 Gy	8.6 ± 1.7 Gy
**Contrl. breast**				
Dmean	5.1 ± 1.9 Gy	4.9 ± 1.9 Gy	5 ± 1.9 Gy	5 ± 1.9 Gy

Hypofractionated radiotherapy (n = 5)	Planning-CT	Estimated delivered dose distribution		
**Heart**				
Dmean	3.5 ± 0.9 Gy	3.1 ± 0.6 Gy	3.1 ± 0.6 Gy	3.1 ± 0.7 Gy
V10Gy	4.8 ± 4%	3.5 ± 3.2%	3.5 ± 3.3%	3.7 ± 3.5%
**LAD**				
Dmean	7.4 ± 5.7 Gy	5.7 ± 3.8 Gy	5.6 ± 3.7 Gy	5.7 ± 3.9 Gy
**Ipsi. lung**				
Dmean	11.2 ± 2.6 Gy	10.7 ± 3.5 Gy	10.6 ± 3.5 Gy	10.7 ± 3.6 Gy
V20Gy	18.4 ± 7.1%	16.3 ± 9.1%	16.2 ± 9.1%	16.2 ± 9.3%
**Total lung**				
Dmean	7.3 ± 1.2 Gy	6.9 ± 1.8 Gy	6.8 ± 1.8 Gy	6.8 ± 1.8 Gy
**Contrl. breast**				
Dmean	3.9 ± 2 Gy	3.6 ± 1.8 Gy	3.6 ± 1.8 Gy	3.5 ± 1.8 Gy

Mean values and standard deviation.

*Significantly different from daily CBCT (*p* < 0.05).

## Discussion

Our results indicate that CBCT imaging significantly reduces the setup error compared to laser-assisted positioning. Nevertheless, image guidance with CBCT on EOD during SIB-irradiation provides similar dose coverage in the target volumes and the OAR compared to daily CBCT. Even weekly CBCT led to only small absolute dose differences in the target volumes and the OAR.

Recommendations on imaging frequency in adjuvant breast cancer radiotherapy are sparse. The NCCN guidelines recommend weekly imaging, but suggest that under “certain circumstances”, more frequent imaging might be appropriate^15^. Despite these suggestions, daily imaging is still widely used in previous studies and daily practice^6,16–18^. This emphasizes the need for further studies that allow more specific recommendations, especially in case of SIB irradiation, since previous studies examining the optimal use of IGRT during adjuvant breast cancer irradiation neglected this technique^8,9^. At the same time, studies that focused on SIB irradiation lacked details on the methods of IGRT. Donovan et al.^16^ performed a phantom study in order to elaborate an image-guided verification protocol for integrated boost treatments in breast cancers. The suggested protocol allows for a PTV-margin of 5 mm and optimization of the dose to the contralateral breast. However, the subject of CBCT imaging frequency was not addressed.

Even though the CBCT imaging dose in most OAR is lower than the dose resulting from VMAT itself, it causes an additional risk of secondary malignancies^19^. Kim et al.^20^ assessed the radiation-induced cancer life-time risk due to kV-CBCT: For female patients undergoing breast cancer radiotherapy in 30 fractions including CBCT to the chest, an additional risk for secondary malignancies due to imaging dose of 7.7/10.000 was estimated. However, the published data assessing CBCT dose during breast cancer treatments varies widely^21,22^. Either way, given the large number of patients treated with adjuvant radiotherapy and the comparatively good prognosis of breast cancer, the additional risk caused by image-guidance needs to be kept as low as reasonably achievable. The use of daily CBCT must therefore be justified by reliable data on the effect of IGRT.

The workflow implemented in the current study allows to quantify the effect of CBCT frequency on the delivered dose. Previous studies consistently reported that CBCT based dose recalculation using CBCT site specific calibration provides highly accurate dose estimations^23,24^, which amplifies the validity of our data. DIR and dose accumulation on the other hand are associated with uncertainties which might impact our results. The estimated dose distribution does not necessarily reflect the actual dose distribution during the treatment using the different CBCT-protocols. However, the technique has been evaluated in previous studies^25,26^ and is also being used in commercially available software, which is approved for being used as medical device (e.g. MIM Software, Inc., Cleveland, Ohio). Overall, the techniques used in the current study can therefore be considered as gold standard for retrospective 3D-dose estimation.

It should be noted that the marker position in our study was not adjusted throughout the treatment, in order to facilitate the interpretation of our results. Adjustment of the marker position based on the CBCTs acquired during the treatment might lead to even better results, which further emphasizes the conclusion of our study.

The most important rational for daily CBCT imaging during SIB irradiation in VMAT technique is the assurance of dose coverage in the SIB-CTV. Our results indicate that adequate dose coverage in the SIB-CTV can also be achieved using CBCT on alternate days or even weekly CBCT, even though the CTV-PTV margins for the tumor bed were as small as 5 mm. Thus, the use of SIB irradiation does not generally demand daily CBCT-imaging.

A further factor justifying the use of daily image guidance is the additional dose to the OARs resulting from insufficient position control. So far, however, only few studies focused on IGRT and dose to the OARs: Lin et al.^27^, demonstrated in a retrospective study on 458 patients that IGRT reduces the occurrence of acute radiodermatitis compared to conventional IMRT. Lemaski et al.^28^ conducted a literature review revealing the potential of IGRT to reduce the dose to the heart during breast cancer radiotherapy. A further study by Basaula et al.^18^ investigated the risk and benefits of target volume margins in breast cancer radiotherapy. The authors created three treatment plans with different target volume margins and assessed the dose to the OARs caused by PTV margin variation and frequency of CBCT (weekly vs. daily). The authors concluded that despite the additional dose from kV-CBCT imaging, smaller PTV margins would result in an overall risk reduction for secondary malignancies. Even though these studies clearly indicate the importance of IGRT for OAR sparing, none of the studies adequately considered the impact of CBCT frequency. Our data reveals that a reduction of CBCT frequency from daily to EOD results in only small dose differences in the OARs. Even weekly CBCT resulted in an only limited increase of OAR dose.

The frequent use of CBCT clearly reduces the step-up errors throughout the treatment (Fig. 2). Yet, the magnitude of couch corrections is an insufficient surrogate to compare the efficiency of different IGRT methods. Instead, the dose distribution in the CTV and the OARs needs to be assessed, since they represent the clinically relevant parameters. In our study, the differences in dose distribution depending on the IGRT frequency were lower than expected. This can be attributed to the effect of safety margins and to the fact that CBCT-based couch corrections do not compensate for all kinds of positioning inaccuracies and anatomical changes.

The limited impact of CBCT frequency on both, target dose coverage and dose distribution in the OARs raises the question, whether alternative methods of IGRT (e.g. daily use of body surface scanners) or the combination of different IGRT methods (2D imaging, CBCT and surface scanners) would suffice for position control during SIB irradiation. This question needs to be addressed in the future. In the meantime, according to our results and even though daily CBCT remains the gold-standard for position control, it can be replaced by CBCT on EOD without compromising target dose coverage or causing a critical dose increase in the OARs.

Given the large effort made in recent years to reduce the dose in the OARs through irradiation in DIBH, improved target volume definition and optimized treatment plans, minimizing the imaging dose seems particularly important. In addition, a reduction of CBCT frequency does not only lower the imaging dose, but also reduces treatment costs, which is an important aspect especially in low income countries.

All patients treated in this study received VMAT, which provides a better PTV homogeneity and a lower dose to the OARs in breast cancer patients treated with RNI and SIB compared to 3D-plans^29^. Nevertheless, many patients are still treated with 3D-CRT and some centers use hybrid techniques combining tangential field irradiation and VMAT^30^. Our results are of only limited validity for these techniques, as the isodose distribution, especially in the heart and lungs, differ significantly between the techniques. This needs to be considered when interpreting our results. Plus, most patients included in our study (n = 45), in accordance with current guidelines for irradiation of the lymph node areas and SIB irradiation^15,31^, were treated in 28 fractions. Yet, several studies investigate the safety and feasibility of SIB irradiation during hypofractionated radiotherapy in only 16 fractions and promising results have already been published^32,33^. Even though the subgroup of patients undergoing hypofractionated radiotherapy with SIB in our study was small (n = 5), our results indicate that the effect of different IGRT protocols was even lower for hypofractionated radiotherapy with SIB due to the reduced number of total fractions, which amplifies our conclusions.

## Conclusion

Compared to the EOD strategy, the improved positioning accuracy through daily CBCT imaging does not result in significant improvement of target dose coverage, nor in significant dose reduction in the OAR. Even with SIB irradiation, the CBCT-frequency should therefore be determined on a case-by-case basis, instead of using daily CBCT as the standard procedure.
